# The Association of the Hypothalamic-Pituitary-Adrenal Axis with Appetite Regulation in Children with Fetal Alcohol Spectrum Disorders (FASDs)

**DOI:** 10.3390/nu15061366

**Published:** 2023-03-11

**Authors:** Rafał Podgórski, Sabina Galiniak, Artur Mazur, Agnieszka Domin

**Affiliations:** 1 Department of Biochemistry, Institute of Medical Sciences, Medical College of Rzeszow University, Warzywna 1a, 35-310 Rzeszow, Poland; sgaliniak@ur.edu.pl; 2 Department of Pediatric, Institute of Medical Sciences, Medical College of Rzeszow University, Warzywna 1a, 35-310 Rzeszow, Poland

**Keywords:** fetal alcohol spectrum disorders, FASD, proopiomelanocortin, ACTH, cortisol

## Abstract

Prenatal alcohol exposure causes growth impairment and a wide range of developmental, physical, and cognitive disorders in children, collectively referred to as fetal alcohol spectrum disorders (FASDs). In the course of FASDs, abnormalities can also affect eating behavior and nutritional status, but these problems have received little attention. Therefore, the aim of our study was to determine the levels of hormones involved in the action of the hypothalamic–pituitary–adrenal axis: proopiomelanocortin (POMC), cortisol, and adrenocorticotropic hormone (ACTH), in the serum of patients with FASDs. To our knowledge, none of these hormones studied have yet been evaluated in FASDs to date. We investigated 62 FASD patients and 23 healthy controls by applying an enzyme-linked immunosorbent method (ELISA). Fasting POMC levels were significantly lower in patients with FASDs (10.97 vs. 18,57 ng/mL, *p* = 0.039) compared to controls. However, there were no differences in cortisol concentrations. Additionally, the sex and subgroup status (fetal alcohol syndrome (FAS), neurobehavioral disorder associated with prenatal alcohol exposure (ND-PAE), and FASD risk) did not affect hormone levels. POMC was positively correlated with some clinical parameters such as age, BMI percentile, carbohydrate biomarkers, and ACTH. A positive correlation was observed between ACTH and cortisol levels, as well as ACTH and cholesterol levels. Data analysis showed no HPA axis abnormalities in the form of elevated serum cortisol and ACTH levels. Differences in POMC concentration may indicate the involvement and/or impairment of central nervous system structures in hormonal alterations in FASD individuals, caused by prenatal alcohol exposure. Hormonal dysregulation in FASDs can contribute to reduced growth and development, as well as many other disturbed processes, including neurological/neurodevelopmental dysfunctions. Further insightful studies involving a larger group of patients are needed to determine the potential impact of the measured hormones.

## 1. Introduction

Exposure to alcohol during the fetal period can disrupt the development of the child and cause a wide range of neurobehavioral disorders, collectively called fetal alcohol spectrum disorders (FASDs). Among them, four distinct diagnostic entities can be distinguished: fetal alcohol syndrome (FAS), partial fetal alcohol syndrome, alcohol-related neurodevelopmental disorder, and alcohol-related birth defects [1]. The harmful effects of alcohol on fetal development were first reported by Jones et al. in 1973 [2]. Since then, despite the growing awareness of the issue in both the medical community and the general public, the problem of fetal exposure to alcohol has remained a serious public health concern. The global prevalence of alcohol consumption during pregnancy between 1984 and 2014 was estimated at 9.8%, but in some countries, such as Australia, New Zealand, and the United Kingdom, the figures are shockingly high and range from 40% to 80% [3,4]. The safe amount of alcohol allowed during pregnancy is not known; therefore, to avoid the risk of FASDs, complete abstinence is recommended. Furthermore, many individuals with a history of prenatal alcohol exposure do not exhibit the characteristic physical features of FASDs and, as a result, may remain undiagnosed or misdiagnosed despite significantly impairing cognitive and behavioral deficits [5]. It should be mentioned that although FASDs are a serious health problem worldwide, to date, no unified diagnostic criteria or classification systems have been developed. The first guidelines were published by the Institute of Medicine in 1996. Currently, commonly used classification systems for FASDs include the four-digit diagnostic code criteria of fetal alcohol spectrum disorder, the Canadian guidelines, and the criteria published by Hoyme et al. in 2016 [6,7,8,9]. Each takes a different approach to assessing the four most important domains, namely the amount and quality of alcohol exposure during pregnancy, impairment of pre- and postnatal growth dysmorphic facial features, and neurodevelopmental abnormalities, and in many respects, they diverge. The clinical symptoms of FASDs comprise a variety of symptoms, including minor craniofacial anomalies, growth retardation, neurological disorders, cognitive and behavioral impairments, and birth defects [6]. The abnormalities can also affect eating behaviors and nutritional status, but these issues have received little attention [10]. Improper eating patterns are common in children with FASDs and may contribute to their growth deficiency and inadequate nutrition [11]. In infancy and early childhood, FASDs are strongly associated with lower-than-normal weight, height, and head circumference [12]. These differences diminish in later childhood, and even increased weight gain is noted compared to the normal population [13,14,15]. The regulation of appetite and satiety is a very complex process that is controlled at the central nervous system (CNS) level by appetite centers in the hypothalamus and brain stem, and peripherally by hormonal signals of energy status released by the intestines and adipose tissue [16]. One of the hormones involved in the regulation of appetite in the CNS is the anorexigenic hormone proopiomelanocortin (POMC), which is a precursor polypeptide yielding several products such as adrenocorticotropic hormone (ACTH); α-, β-, and γ-melanocyte-stimulating hormones (α-MSH, β-MSH, and γ-MSH); β-lipotrophin; and endorphins [17,18]. The pituitary-derived ACTH regulates cortisol secretion via the adrenal cortex by binding to the adrenal melanocortin receptors (MC2Rs) [17]. Cortisol has important regulatory effects throughout the body and the brain, affecting the response to stress, energy and metabolic processes, immune and inflammatory function, and mood and sexual behavior [19].

The goal of the current study was to determine the levels of POMC, ACTH, and cortisol involved in the hypothalamic–pituitary–adrenal (HPA) axis as well as the regulation of appetite in patients diagnosed with FASDs and to compare these with a control group. To our knowledge, this is the first study to describe such an issue in patients with FASDs.

## 2. Materials and Methods

Study group. A single-center, cross-sectional study was conducted on 62 FASD patients aged 4 months to 16.5 years and 23 healthy controls. The group of patients was divided into 3 subgroups: FAS, ND-PAE (neurobehavioral disorder associated with prenatal alcohol exposure), and FASD risk. FASDs were diagnosed according to the latest guidelines of the Polish recommendations [20]. With respect to the internationally developed guidelines applicable to the Polish context, the ND-PAE domain includes partial fetal alcohol syndrome and alcohol-related neurodevelopmental disorder [6,7,8,9]. Participants were recruited from the Department of Pediatrics, Pediatric Endocrinology and Diabetology, and the Endocrinology Outpatient Clinic. The study protocol was approved by the Bioethics Committee of Rzeszow University (16 February 2019). All the procedures performed in studies involving human participants were in accordance with the ethical standards of the institutional and/or national research committee and with the Declaration of Helsinki of 1964 and its subsequent amendments or comparable ethical standards. Informed consent was obtained from all participants or if they were under 16 years of age, from a parent and/or legal representative.

Blood sampling. Blood samples for POMC and cortisol determination were collected in the morning between 8:00 and 10:00 a.m., fasting. Next, blood was incubated at room temperature for at least 30 min and centrifuged (1500× *g*, 10 min, 4 °C). Subsequently, the serum was transferred to cryovials and stored in a freezer at −80 °C until further analysis. The determination of ACTH was performed in a blood sample collected into a cooled plastic tube containing EDTA. The tubes were then placed in ice water, immediately delivered to the laboratory, centrifuged (1500× *g*, 10 min, 4 °C), and analyzed.

Determination of hormone levels. proopiomelanocortin (POMC) serum concentrations after an overnight fast were measured in duplicate with previous dilution using commercially available enzyme-linked immunosorbent assays (Wuhan Fine Biotech Co., Ltd., Wuhan, China), according to the manufacturer’s protocol. The limit of detection for POMC was 0.094 ng/mL, and the within- and between-assay coefficients of variations were lower than 8% and lower than 10%, respectively. Cortisol and adrenocorticotropic hormone (ACTH) were determined using an Alinity analyzer (Abbot, Abbott Park, IL, USA) using the chemiluminescent microparticle immunoassay method. Blood morphology was analyzed using a hematology analyzer (Siemens Healthineers, Germany). Other clinical parameters were obtained from patients’ clinical records.

Statistical analysis. All statistical analyses were performed applying the STATISTICA software package (version 13.3, StatSoft Inc. 2017, Tulsa, OK, USA). Data were expressed as mean and SD or median, as well as range. Most variables did not follow a normal distribution, which was validated using the Shapiro–Wilk test, due to the non-parametric tests that were applied. The Mann–Whitney U test was used for comparison between two independent groups, and for multiple comparisons, the Kruskal–Wallis ANOVA was used. A *p*-value below 0.05 was considered statistically significant. The correlation analysis was performed using the Spearman correlation test, assuming linear dependence with α = 0.05.

## 3. Results

A total of 62 affected individuals with FASDs were recruited into the study, including 31 boys and 31 girls. At the same time, 23 healthy children, including 16 boys and 7 girls (30.5%), were enrolled in the study. The basic laboratory and anthropometric parameters of patients with FASDs and healthy controls are presented in Table 1.

There were no age differences between patients with FASDs and healthy controls. We found a significant difference in age between the subgroups within FASDs (Table 2), as a result of the lower age of the FASD risk group, but the differences between the FAS and ND-PAE groups were not statistically relevant (*p* = 0.843). BMI percentile values were significantly lower in the FASD group than in the control group (*p* = 0.035). Differences in BMI were also observed between FASD subgroups. The BMI in the ND-PAE group was higher than in the FAS group (*p* = 0.020).

In the FAS subgroup, children with low height (<3 percentile) accounted for 42.31%, and the majority of children with body heights below the third percentile were girls (90.91%) compared to boys (9.09%). In the ND-PAE subgroup, children with low height comprised 18.18%, with the largest group being patients between the 25th and 50th percentile (27.27%), with a frequency of 50% in both girls and boys. In the FASD RISK group, low-growth children accounted for 20%. There were no significant differences between the compared groups in biochemical parameters such as lipid profile, glucose, insulin levels, or HOMA-IR.

**Figure 1:**
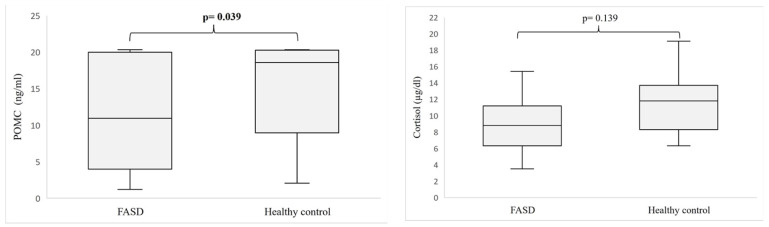
Level of POMC and cortisol in patients with FASDs compared to healthy participants. Abbreviations: POMC—proopiomelanocortin; FASD—fetal alcohol spectrum disorder. Comparisons between means were analyzed using Mann–Whitney U test. Statistically significant differences are in bold.

The levels of the hormones analyzed are shown in Figure 1. The POMC level was significantly decreased in the serum of patients with FASDs compared to healthy individuals (*p* = 0.039).

However, a comparison of POMC levels between FASD subgroups showed no significant differences (Table 3), indicating that reduced POMC secretion may be associated with prenatal alcohol exposure. Cortisol concentrations were also lower in individuals with FASDs, but the differences were not statistically significant. ACTH was determined only in FASD participants, and we found no discrepancies in ACTH and cortisol levels between FASD subgroups.

**Figure 2:**
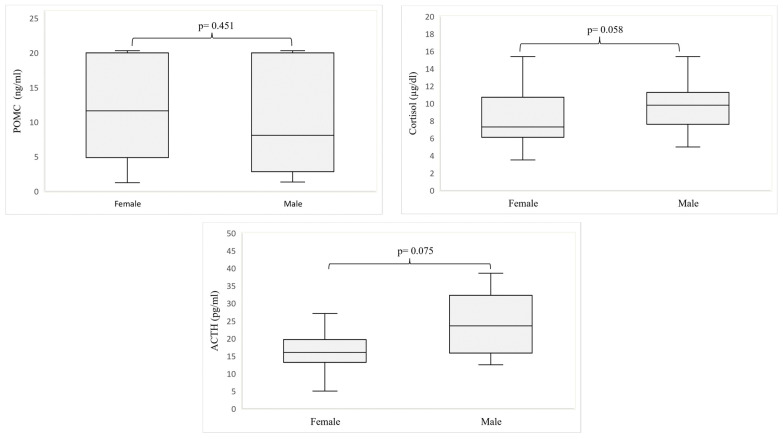
Hormone levels by sex of patients with fetal alcohol spectrum disorder. Abbreviations: POMC—proopiomelanocortin; ACTH—adrenocorticotropin. Comparisons between means were analyzed using Mann–Whitney U test.

Figure 2 presents a comparison of the hormone concentrations analyzed in girls and boys with FASDs. We did not observe any difference in hormone levels between girls and boys affected by FASDs, but cortisol and ACTH concentrations were higher in boys, and these differences were close to statistical significance (0.058 and 0.075, respectively).

The next step in data analysis was to evaluate the correlation between the levels of the hormones studied and the clinical parameters of patients with FASDs (Table 4). POMC was positively correlated with age and BMI percentile (R = 0.370, *p* = 0.003; R = 0.285, *p* = 0.030, respectively). The parameters describing carbohydrate metabolism and regulation, such as HOMA-IR, glucose, and insulin levels, were positively associated with POMC levels (R = 0.473, *p* < 0.001; R = 0.292, *p* = 0.026; R = 0.475, *p* < 0.001, respectively). We found no correlation between ACTH, cortisol, and patients’ clinical parameters, with the exception of cholesterol, which showed a positive correlation with ACTH (R = 0.335, *p* = 0.046). In addition, the correlations between the hormones studied were analyzed, which showed a strong positive association between ACTH and cortisol (R = 0.607, *p* < 0.001) and a moderate positive correlation between POMC and ACTH (R = 0.386, *p* = 0.020), while no correlation was found between POMC and cortisol levels. POMC was also not correlated with the parameters of the lipid profile.

## 4. Discussion

Our study describes, for the first time, the circulating levels of hormones involved in the function of the HPA axis and regulation of energy metabolism and nutrition such as proopiomelanocortin, adrenocorticotropic hormone, and cortisol in patients with fetal alcohol spectrum disorders. The comparison with the healthy controls revealed a significantly lower concentration of POMC in patients with FASDs, whereas there were no differences in cortisol levels. FASDs manifest in different ways, including height and weight deficiency, inappropriate eating patterns, and nutritional deficits [11]. POMC is a prohormone secreted via the pituitary, hypothalamus, medulla, and several peripheral tissues and plays a key role in the regulation of energy balance and neuroendocrine function. POMC is transformed in a tissue-specific manner to produce biologically active peptides, among others, ACTH, α-MSH, or the opioid peptide β-endorphin [21]. α-MSH is produced in the arcuate nucleus of the hypothalamus and activates melanocortin receptors (MC4 and MC3) to regulate energy balance by stimulating energy expenditure and inhibiting feeding. The opposite orexigenic effects and reduction in energy expenditure are induced by Agouti-related protein (AGRP) [22]. These hormones and receptors form the hypothalamic melanocortin system, responsible for regulating energy homeostasis by affecting feeding behavior and energy expenditure [23]. POMC can reduce appetite and food intake on both immediate and longer time scales [24]. Several studies have shown that the inactivation of the melanocortin system or POMC deficiency in animal and human models results in obesity and insulin resistance [25,26,27]. Our study showed a decrease in POMC levels in patients with a spectrum of fetal alcohol disorders compared to the control group, but there were no differences between FASD subgroups, namely FAS and ND-PAE subgroups. These outcomes may indicate that POMC is an important contributor to poor weight gain and growth development or neurodevelopmental abnormalities in individuals with FASDs. In a rat model, POMC neurons have been reported to be killed as a result of developmental alcohol exposure due to the activation of microglial immune cells in the brain [28]. The dysregulation of the POMC system function due to prenatal alcohol exposure is induced by some epigenetic mechanisms such as hypermethylation of the POMC gene promoter and an alteration in histone marks in POMC neurons [29]. Moreover, the epigenetic modifications of the POMC gene can be passed down through the generations via the male germline and may be strongly involved in alcoholism-inherited disorders [30]. These findings may explain the lowered POMC secretion in those with FASDs as a result of the degenerative effects of alcohol on POMC neurons during the prenatal period. To date, there are only a few reports on the determination of POMC in serum using the immunoenzymatic method. Our recent study in cystic fibrosis patients showed reduced POMC levels compared to controls [31]. On the other hand, patients with anorexia nervosa have shown significantly higher levels of POMC than controls [32]. POMC plasma level was determined as a potential differential marker of ACTH-dependent Cushing’s syndrome [33]. POMC level was also measured using the ELISA technique in the cerebrospinal fluid of obese individuals and was higher in lean subjects [34].

Furthermore, POMC neurons are known to regulate not only energy homeostasis but also the hypothalamic–pituitary–adrenal axis and the immune system [35]. Recent studies have shown that early long-term gestational alcohol exposure alters HPA axis activity [36]. Prenatal alcohol exposure programs the hypothalamus to produce lower levels of POMC gene transcripts and increases the response to stressful factors [37]. The HPA axis under physiological conditions allows the human body to adapt and respond to physical or emotional stressors and maintain homeostasis [38]. The hypothalamus secretes corticotropin (CRH), which in turn stimulates the pituitary gland to release various POMC-derived peptides, including adrenocorticotropin. ACTH regulates the growth and activity of the adrenal cortex and induces the secretion of glucocorticoids, the most important representative of which is cortisol. The glucocorticoids secreted into the bloodstream affect the pituitary gland, the hypothalamus, and other brain structures, resulting in the inhibition of the HPA axis in a process called a negative feedback loop [39,40]. In response to short-term stress stimuli, glucocorticoids mobilize the body’s energy resources at the expense of energy-dependent functions—digestion, growth, and reproduction. When the stressful situation is prolonged or occurs frequently, the metabolic effects that occur and the redistribution of energy resources can have pathological consequences, including gastrointestinal disorders (mucosal ulcerations), growth retardation or low stature in children, and impaired immune system responses [41,42]. The consumption of ethanol by the mother during pregnancy activates the HPA axis in her organism and in the fetus. As a result, glucocorticoid levels increase. The functioning of the entire HPA axis and the feedback loop is disrupted. Several studies have shown excessive cortisol secretion in response to a stressful stimulus, as well as the disturbed diurnal rhythms of secretion [43,44,45]. The dysregulation of the HPA axis may contribute to some of the cognitive, behavioral, and adaptive disorders observed in people with FASDs, as well as their susceptibility to mental health and sleep disorders [44,46]. Studies in children with FASDs showed that they had significantly higher levels of cortisol in the afternoon and evening and, as in our results, a tendency towards lower levels in the morning compared to controls, suggesting a possible disruption of normal basal HPA regulation during the day [44]. In our study, we found no differences in cortisol levels in FASD individuals compared to controls, nor in FASD subgroups or between males and females. Similar results were observed in infants prenatally exposed to cigarettes, who showed no difference in cortisol levels prior to exposure to stressful stimuli but revealed a higher cortisol response than unexposed infants [47]. However, decreased cortisol reactivity has also been described [45]. Alterations in cortisol diurnal rhythm secretion (elevated afternoon and evening cortisol levels) seem to be important factors leading to neurological disruptions such as depression, PTSD, and sleep disorders [44,48]. There is also strong evidence that cortisol promotes eating. Several studies demonstrated that participants with higher cortisol levels ate more food versus controls [49,50]. Cortisol increases appetite by decreasing brain sensitivity to leptin, regulating orexigenic hormone–neuropeptide Y stimulation, and potentiating reward pathways [51,52]. Furthermore, cortisol directly promotes fat deposition, particularly in the abdominal region, in a mechanism observed in Cushing’s disease [53,54]. In our study, FASD individuals showed reduced cortisol levels compared to controls, but these differences were not statistically significant. The lack of differences in cortisol levels and the identified HPA axis disorders in our study may be the result of the time of blood collection, as morning cortisol levels are usually not elevated, and changes might be observed after evening sampling.

In contrast to cortisol, the intracerebroventricular or intraperitoneal injection of ACTH has been reported to decrease food intake [36,55]. Furthermore, in animal models, alcohol induces ACTH secretion [56]. The administration of MSH/ACTH (4–10) analogues in normal-weight humans reduced body fat, body weight, and plasma insulin level [57]. These effects were not observed in patients with POMC mutations [58]. Recent studies in rodents also revealed that prenatal alcohol exposure causes the dysfunction of the HPA axis, showing elevated levels of corticosterone (the animal equivalent of cortisol) and ACTH after exposure to stress factors such as acute alcohol injection [59]. These findings seem not to be confirmed in our study because cortisol levels did not differ from control levels. FASD severity and sex had no effect on the concentrations of POMC, ACTH, and cortisol.

The correlations between the hormone levels studied and the clinical parameters of the patients with FASDs were also evaluated. To our knowledge, no studies have investigated the correlation between POMC, ACTH, cortisol, and clinical data in FASD patients. We found a positive correlation of POMC with age, BMI percentile, and ACTH levels. Similarly to our results, the POMC level of cerebrospinal fluid was positively correlated with ACTH but negatively with BMI and adiposity in overweight and obese patients [34]. However, cerebrospinal fluid and plasma POMC were not correlated [34]. Contrary to our results, a significant negative correlation was also observed between serum POMC levels and BMI (R  = −0.526, *p* < 0.010) in subjects with anorexia nervosa [32]. These contradictory outcomes may indicate that the mechanism of POMC excretion mechanism is not directly related to the content of adipose tissue, but its regulation is more complicated. Furthermore, in this report, POMC was positively correlated with carbohydrate metabolism makers and regulators such as glucose, HOMA-IR, and insulin. Contradictory results were observed in the cerebrospinal fluid of overweight patients with respect to insulin and HOMA-IR (R = −0.33, *p* = 0.03; and R = −0.20, *p* = 0.23, respectively) [34]. The influence of insulin on POMC gene expression was investigated. Insulin activates the phosphatidylinositol 3-kinase signaling cascade, leading to the inhibition of nuclear forkhead box protein O1, neuronal membrane hyperpolarization, and electrical silencing, resulting in the increased expression of POMC mRNA [60]. Similarly to previous results, we observed a markedly strong positive correlation between cortisol and ACTH levels, but simultaneously cortisol was not correlated with POMC, whereas ACTH was [61,62]. Cortisol secretion after ACTH stimulation was applied in clinical practice to assess the function of the HPA axis in patients with adrenal dysfunction due to pituitary, hypothalamic, or adrenal diseases [63]. Cortisol and ACTH were also not correlated with age, sex, and BMI percentile, as in previous studies in healthy children [64,65,66]. However, conflicting results have also been described, especially in young children under the age of eight. The lack of differences at a later age is probably the result of the sex hormones on the hepatic metabolism of cortisol [67,68]. Carbohydrate metabolism markers in individuals with FASDs were not correlated with ACTH and cortisol levels, but a positive association with fasting glucose (R = 0.193, *p* < 0.005) and triglycerides (R = 0.143, *p* < 0.05) were noticed in children and adolescents with overweight and obesity [66].

Finally, we did not find any correlation between the hormones studied and markers of the lipid profile, except for a moderate association between ACTH and cholesterol levels, which may indicate that these factors are not predictors of serum levels of these hormones in patients with FASDs.

The presented study provides, for the first time, interesting and valuable information on the effects of prenatal alcohol exposure on the hormonal regulation of nutrition and the HPA axis. However, the results and conclusions provided may be subject to some uncertainty due to the insufficient number of participants. In addition, ACTH levels were determined only in affected subjects, which did not allow comparison with the control group. Furthermore, fluctuations in cortisol and ACTH levels could be recorded more easily in the evening, so an additional sampling period should be added in the future. Finally, an enzymatic immunoassay is prone to cross-reactivity with other biological compounds, and more sensitive and selective methods such as chromatographic techniques or genetic analyses should be implemented to confirm our findings. Our outcomes should be considered as a prelude to further multicenter studies involving a larger group of patients to determine the concentration of POMC, cortisol, and ACTH and their influence on the clinical outcomes of patients with FASDs.

## 5. Conclusions

In conclusion, we investigated 62 patients with FASDs and 23 healthy controls. We found significantly reduced levels of POMC in patients with FASDs. However, there were no differences in cortisol concentrations. Furthermore, we did not find any differences in POMC, ACTH, and cortisol levels between various subgroups of FASDs, i.e., FAS, ND PAE, and FASD RISK. Additionally, sex did not affect hormone levels. In our study, we showed some interesting correlations between the level of hormones and the parameters that describe the clinical condition of patients with FASDs. The analysis of the results showed no HPA axis abnormalities in the form of elevated serum cortisol and ACTH levels, without the use of stress stimuli other than those associated with blood draws. Differences in POMC concentration may indicate the involvement and/or impairment of central nervous system structures in hormonal alterations in FASD individuals, caused by prenatal alcohol exposure. Hormonal dysregulation in those with FASDs can contribute to reduced growth and development, as well as many other disturbed processes, including neurological dysfunctions; therefore, further studies are needed to elucidate these aspects.

## Figures and Tables

**Table 1:** Baseline demographic and clinical data of the study participants.

		FASD	Healthy Controls	*p-*Value
Sex (F/M)		31/31	7/16	
Age (years)	mean ± SD	7.52 ± 4.16	7.45 ± 5.12	0.847
	range	0.42–16.5	0.42–17	
BMI percentile	mean ± SD	32.38 ± 31.24	60.71 ± 27.03	**0.035**
	range	0.1–99.9	12.0–99	
Clinical laboratory markers				
Cholesterol (mg/dL) Norm < 190	median	150	155	0.971
	range	76–244	126–191	
LDL (mg/dL) Norm < 135	median	90	95	0.827
	range	31–163	72–104	
HDL (mg/dL) Norm > 40	median	53	53	0.856
	range	24–108	42–59	
Triglycerides (mg/dL) Norm < 150	median	74	65	0.753
	range	30–241	38–141	
Glucose (mg/dL) Norm (70–99)	median	84	87	0.669
	range	72–99	68–94	
Insulin (mIU/mL) Norm < 15	median	4.85	2.05	0.167
	range	1.25–17	1.0–9.03	
HbA1c (%) Normal range (4–6)	median	5.36	5.41	0.774
	range	4.71–5.86	5.26–5.55	
HOMA-IR Norm < 2.5	median	0.96	-	-
	range	0.23–3.62	-	

Abbreviations: BMI percentile—body mass index percentile; LDL—low-density lipoprotein; HDL—high-density lipoprotein; HbA1c—glycated hemoglobin; HOMA-IR—homeostasis model assessment of insulin resistance. Data are presented as mean and SD or median and range; differences between means were analyzed using Mann–Whitney U test. Statistically significant differences are in bold.

**Table 2:** Baseline demographic and clinical data of FASD subgroups.

		FAS	ND-PAE	FASD Risk	*p-*Value	*p-*Value *
Sex (F/M)		14/12	15/16	2/3		
Age (years)	mean ± SD	7.91 ± 4.77	8.13 ± 3.32	2.25 ± 1.26	**0.004**	0.843
	range	0.42–16.5	2.08–13.5	1.17–4.42		
BMI percentile	mean ± SD	22.12 ± 27.51	42.04 ± 33.02	27.33 ± 13.87	0.053	**0.020**
	range	0.1–78	0.1–99.9	12–39		
	Clinical laboratory markers					
Cholesterol (mg/dL) Norm < 190	median	154.5	161	141	0.277	0.110
	range	76–238	114–244	104–185		
LDL (mg/dL) Norm < 135	median	86	75	84	0.598	0.365
	range	31–143	114–244	33–119		
HDL (mg/dL) Norm > 40	median	49.5	53	46	0.515	0.382
	range	33–80	24–108	33–71		
Triglycerides (mg/dL) Norm < 150	median	64	75	75	0.590	0.607
	range	30.0–229	34–241	55–99		
Glucose (mg/dL) Norm (70–99)	median	82	87	80	0.111	0.211
	range	72–99	74–99	76–91		
Insulin (mIU/mL) Norm < 15 mIU/mL	median	5.10	4.22	3.22	0.596	0.623
	range	1.41–16.46	1.56–13.97	1.25–17		
HbA1c (%) Normal range (4–6)	median	5.24	5.45	5.35	0.076	**0.039**
	range	4.81–5.86	4.89–5.85	4.71–5.53		
HOMA-IR Norm < 2.5	median	1.07	1.29	1.73	0.591	0.790
	range	0.27–3.62	0.31–3.53	0.23–3.36		

Abbreviations: BMI percentile—body mass index percentile; LDL—low-density lipoprotein; HDL—high-density lipoprotein; HbA1c—glycated hemoglobin; HOMA-IR—homeostasis model assessment of insulin resistance. Data are presented as mean and SD or median and range; comparisons between means were analyzed using Kruskal–Wallis test or * Mann–Whitney U test (comparison between FAS and ND-PAE only). Statistically significant differences are in bold.

**Table 3:** Hormone levels in subgroups of patients with FASDs.

Hormone		FAS	ND-PAE	FASD Risk	*p-*Value	*p-*Value *
POMC (ng/mL)	median	9.35	15.93	11.22	0.725	0.479
	range	1.22–20.32	1.51–20.32	3.92–16.84		
ACTH (pg/mL)	median	24.9	17.7	14.3	0.942	0.401
	range	12.3–38.6	5.0–37.6	12.5–66.1		
Cortisol (µg/dL)	median	9.9	7.95	9.0	0.649	0.370
	range	4.30–18.9	3.5–26.0	6.1–23.2		

Data are presented as median and range; comparisons between means were analyzed using Kruskal–Wallis test or * Mann–Whitney U test (comparison between FAS and ND-PAE only).

**Table 4:** Spearman rank correlation coefficients (R) and *p*-values between hormone concentrations and clinical features of the patients studied.

		Age	BMI Percentile	Cortisol	ACTH	Cholesterol	LDL	HDL	TGL	Glucose	Insulin	HOMA-IR	HbA1c	POMC
POMC	*R*	0.370	0.285	0.015	0.386	0.193	0.126	0.154	0.177	0.292	0.475	0.473	0.076	
	*p*	**0.003**	**0.030**	0.912	**0.020**	0.137	0.333	0.236	0.173	**0.026**	**<0.001**	**<0.001**	0.587	
ACTH	*R*	0.304	0.306	0.607		0.335	0.262	0.208	0.175	0.152	0.183	0.189	0.045	0.386
	*p*	0.071	0.083	**<0.001**		**0.046**	0.141	0.245	0.33	0.398	0.307	0.292	0.805	**0.020**
Cortisol	*R*	0.071	−0.093		0.607	0.106	0.122	−0.024	0.188	−0.158	−0.089	−0.127	−0.116	0.015
	*p*	0.592	0.497		**<0.001**	0.444	0.379	0.866	0.172	0.268	0.543	0.386	0.424	0.912

Abbreviations: BMI percentile—body mass index percentile; POMC—proopiomelanocortin; ACTH—adrenocorticotropic hormone; LDL—low-density lipoprotein; HDL—high-density lipoprotein; TGL—triglycerides; HOMA-IR—homeostasis model assessment of insulin resistance; HbA1c—glycated hemoglobin. Statistically significant differences are in bold.

## Data Availability

The data presented in this study are available on request from the corresponding author.
